# Comparative genomics of the Rab protein family in Apicomplexan parasites

**DOI:** 10.1016/j.micinf.2008.01.017

**Published:** 2008-04

**Authors:** Gordon Langsley, Vera van Noort, Céline Carret, Markus Meissner, Etienne P. de Villiers, Richard Bishop, Arnab Pain

**Affiliations:** a Laboratoire de Biologie Cellulaire Comparative des Apicomplexes, Département de Maladies Infectieuses, Institut Cochin, Inserm, U567, CNRS, UMR 8104, Faculté de Médecine Paris V – Hôpital Cochin, 27, rue du Faubourg Saint-Jacques, 75014 Paris, France; b Centre for Molecular and Biomolecular Informatics, Nijmegen Centre for Molecular Life Sciences, Radboud University Nijmegen Medical Centre, PO Box 9101, 6500 HB Nijmegen, The Netherlands; c Pathogen Microarrays Group, The Wellcome Trust Sanger Institute, Hinxton, Cambridge CB10 1SA, UK; d Hygieneinstitut Heidelberg, Abt. Parasitologie, Im Neuenheimer Feld 324, 69120 Heidelberg, Germany; e International Livestock Research Institute, PO Box 30709, Nairobi, Kenya; f The Pathogen Sequencing Unit, The Wellcome Trust Sanger Institute, Hinxton, Cambridge CB10 1SA, UK

**Keywords:** *Theileria*, Apicomplexa, Rab GTPases, Vesicular traffic

## Abstract

*Rab* genes encode a subgroup of small GTP-binding proteins within the *ras* super-family that regulate targeting and fusion of transport vesicles within the secretory and endocytic pathways. These genes are of particular interest in the protozoan phylum Apicomplexa, since a family of Rab GTPases has been described for *Plasmodium* and most putative secretory pathway proteins in Apicomplexa have conventional predicted signal peptides. Moreover, peptide motifs have now been identified within a large number of secreted *Plasmodium* proteins that direct their targeting to the red blood cell cytosol, the apicoplast, the food vacuole and Maurer's clefs; in contrast, motifs that direct proteins to secretory organelles (rhoptries, micronemes and microspheres) have yet to be defined. The nature of the vesicle in which these proteins are transported to their destinations remains unknown and morphological structures equivalent to the endoplasmic reticulum and trans-Golgi stacks typical of other eukaryotes cannot be visualised in Apicomplexa. Since Rab GTPases regulate vesicular traffic in all eukaryotes, and this traffic in intracellular parasites could regulate import of nutrient and drugs and export of antigens, host cell modulatory proteins and lactate we compare and contrast here the Rab families of Apicomplexa.

## 1 Introduction

Apicomplexan parasites are a phylum of medically important infectious organisms responsible for a large number of diseases that afflict mankind and its domestic animals. The phylum contains such notable parasites such as *Plasmodium* – *P. falciparum* the causative agent of human malaria; *Toxoplasma* – *T. gondii* the causative agent of Congenital Toxoplasmosis; *Cryptosporidia* – *C. parvum* and *C. hominis* the causative agents of persistent diarrhea; *Neospora* – *N. caninum* the causative agent of abortion in a wide-range of animals; *Babesia* – *B. bovis* the causative agent of Tick fever in cattle and *Theileria* – *T. parva* the causative agent of East Coast fever and *T. annulata* the causative agent of Tropical Theileriosis. Due to their medical importance, the sequence of the genomes of many of these Apicomplexan parasites has been determined, their proteomes compared [1] and transcriptional data exploited [2–5].

Rabs are small GTP-binding proteins that regulate targeting and fusion of transport vesicles within the secretory and endocytic pathways of eukaryotic cells [6]. The sequencing of *T. parva* and *T. annulata* has revealed two highly syntenic genomes with 82% nucleotide identity [7] that we have exploited using comparative genomics to characterise the two families coding for Rab GTPases and then to compare them with Rab families from other Apicomplexan parasites. Given the wealth of information available on *P. falciparum* via PlasmoDB (http://www.plasmodb.org), or GeneDB (http://www.genedb.org) and the fact that the complete family of 11 Rabs has been characterised [8], we have used this family of parasite Rabs as a benchmark for our comparative analysis, particularly with respect to transcription profiling. Moreover, a comparison between *Theileria* and *Babesia* parasites that lack a parasitophorous vacuole membrane (PVM) with *Plasmodia* and *Toxoplasma*/*Neospora* parasites that reside within a PVM might throw some light as to a potential role of a given Rab in mediating vesicular traffic across this barrier [9–11]. We have also included in our analysis *Cryptosporidia*, as these parasites have lost the Apicoplast, an organelle believed to be derived from the chloroplast of an ancestral algal endosymbiont that characterises many Apicomplexan parasites, and to which vesicular traffic is likely to occur [12].

Many microarray studies have been performed using *P. falciparum* parasites and this has led to the notion that its transcriptional regulation is unusual with peaks of gene expression occurring in waves, where genes encoding related functions (such as invasion) are expressed at the same time [3,4,13,14]. The concept of unusual regulation of transcription in Apicomplexa was reinforced by a study using massively parallel signature sequencing (MPSS) of *T. parva* transcripts that showed that polyadenylated transcripts corresponding to 86% of *T. parva* genes had signature sequences in cultured infected lymphocytes harvested at a single time point [15]. Another unusual feature of transcription in Apicomplexa is the abundance of anti-sense transcripts that we will address in detail later. This level of both sense and anti-sense transcripts is consistent with the hypothesis that in Apicomplexa virtually all genes are transcribed at a basal level, but that transcripts for subsets of genes are subject to specific regulatory processes and can accumulate at different points in the life cycle. One way to explain this kind of control is via the recombinatorial binding of different factors to the regulatory regions upstream of coding sequence of *P. falciparum* genes [16], a notion that could explain the dearth of recognisable transcription factors encoded in the genome [1,17]. We have used previously described algorithms [16] to identify putative factor binding motifs in the regulatory regions of *P. falciparum rab* genes and we then compared the presence and position of these motifs to the transcription profiles of the different *rab* genes, as determined from published microarray data [3,4,14].

As we have previously shown, phylogenetic analysis allows grouping of different parasite Rabs into clades [8] and such associations allow us to propose similar putative functions for Rabs from the different Apicomplexa. Unlike *Plasmodia* [8] that have 11 *rab* genes, *Toxoplasma* and *Neospora* (not shown) encode 15 different Rabs probably reflecting their large host range. In contrast, *Theileria* and *Babesia* parasites have a smaller family of 9 Rabs, lacking a gene coding for Rab5A and Rab18 and in *Cryptosporidia* the Rab family is further reduced to only 8, as they also lack Rab5B. This could be taken as suggesting that Rab5A and Rab18 might be involved in vesicular traffic to the PVM, while Rab5B might regulate a trafficking towards the Apicoplast of *Plasmodia* and *Toxoplasma*.

## 2 The *Theileria* family is made up of 9 Rabs two of which exhibit unusual functional properties

To determine the complete complement of *rab* genes encoded in the *T. parva* and *T. annulata* genomes we performed an exhaustive series of BLAST analyses using *P. falciparum rab* genes as queries. In this way we established that both *Theileria* species have just 9 *rab* genes and that they appear to lack orthologues for *rab5b* and *rab18* (Table 1). We noted the detection of a corresponding *T. annulata* expression sequence tag (EST) for each *rab* gene and whether the EST was derived from schizont (infected macrophages) or piroplast (infected red blood cells) mRNA, so as to gain some insight into the expression profile of the *T. annulata rab* family at two different life cycle stages. Even this rather superficial evaluation of expression profiles demonstrates that 7 out of 9 *T. annulata rabs* are expressed in infected macrophages clearly suggesting that all *rabs* are expressed at this stage. The lower number of corresponding piroplast ESTs is probably a reflection of the small size of the piroplast cDNA library [7].

Two *T. parva* Rab proteins have previously been shown to exhibit unusual functional properties [18]. A Rab1B homologue of *T. parva* contained a 17 amino acid C-terminal extension and a novel XCX motif for addition of a lipid moiety through isoprenylation that differed from the typical CXC or XCC signal, but was shown to be functional in vitro [18]. Moreover, immunofluorescence indicated that *T. parva* Rab1B was expressed in schizont-infected lymphocytes with a perinuclear localisation typical for an ER-specific Rab. A second cDNA (GenBank accession number DQ825390) was originally described as being most similar to Rab4 [18]. However, BLAST searches performed against the range of Apicomplexan Rabs as part of this study suggest that it is a Rab11A orthologue. This Rab also contains an unusual sequence feature in having a substitution of Alanine at position 146 located within the third GTP-binding domain motif by a Cysteine. An Alanine residue is typically conserved in this position, not only in Rabs, but also in the entire *ras* super-family. Inspection of the different parasite genome sequences confirmed the substitution of Ala146 residue by Cysteine in *T. annulata* and in *B. bovis*.

## 3 Transcripts of the *T. parva rab* family are present in infected T cells

An alternative estimate of gene transcription can be obtained using massively parallel signature sequencing (MPSS) and this has been applied to *T. parva* using mRNA isolated from infected T cell cultures [15]. We have extracted from this genome-wide data set the MPSS scores for the 9 *T. parva rab* genes (Table 2). One can readily see what we suspected from the *T. annulata*-infected macrophage EST collection, namely that transcripts for 7 different *rab* genes are detected in infected T cells. Although no transcripts were detected for *rab1b* and *rab11b* this is due to the absence of a DpnII restriction site that is required in the MPSS technique in order to clone the relevant cDNAs for generation of sequence signatures [15]. Moreover, ESTs were detected for both *rabs* in *T. annulata*-infected macrophages (Table 1) consistent with the two genes being transcribed. Taking the *T. parva* and *T. annulata* expression profiling data together it seems reasonable to propose that all *Theileria rab* genes are transcribed in infected leukocytes.

An advantage of MPSS is that the score (the number of transcripts sequenced) gives a more readily quantifiable estimate of the level of transcription. The individual scores indicate that different *T. parva rab* genes are transcribed at varying levels, going from a minimum of 4 for *rab1a* to 261 for *rab2*. It should be noted that in general *rab* gene transcription appears low compared to that of another small GTPase like *ran* that has a score 10-fold higher (Table 2). The implied level of transcription is also low relative to *T. parva* genes in general, with the average number transcripts per million (t.p.m) being 232 for sense signatures across the entire genome [15]. This would imply that *rab*-specific promoters, if they exist, are weak in nature.

There is a growing debate as to the potential role of anti-sense transcripts in *P. falciparum*, where they can be detected for approximately 12% of all genes [19,20]. Anti-sense transcription could be a phenomenon common to all Apicomplexa, as a similar percentage of genes have anti-sense transcripts in *T. parva* [15]. Anti-sense transcripts were detected only for *rab11a*, *i.e.* 1 out of 9 *rab* genes (11%), which is in the range described for the whole genome (Table 2). This contrasts with the gene for another GTP-binding protein (TP03_0578) that has 20-fold more anti-sense message. The anti-sense transcripts could not be explained by mRNA coming from another gene being transcribed on the opposite strand of the chromosome. Why there is an abundance of anti-sense transcription in Apicomplexa remains obscure, but it could suggest that transcriptional control in parasites is promiscuous, with the polymerase binding to and transcribing any accessible (chromatin poor) DNA. Another, non-exclusive possibility is that anti-sense transcripts are regulators of gene transcription.

**Fig. 1:**
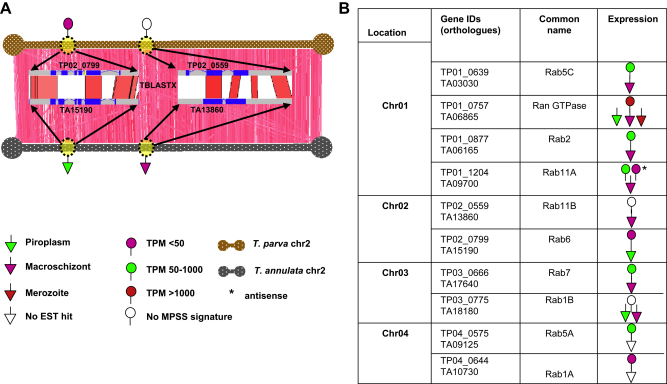
Positionally conserved *rab* genes and their expression profiles in *Theileria* genomes. (A) Artemis comparison tool (ACT) view of conserved gene order (*i.e.* synteny) between *T. annulata* and *T. parva* chromosome 2 is shown. The locations of the Rab6 and Rab11B orthologs within chromosome 2 in *T. annulata* and *T. parva* are indicated with yellow spheres. The red lines connecting the 2 chromosomes represent TBLASTX matches. The annotated gene structures of the Rab6 and Rab11B in *T. annulata* and *T. parva* are shown as the zoomed in view. (B) The expression levels (as determined by the MPSS data or EST data) of the *rab* genes, their chromosomal position and orthologous relationships between *T. annulata* and *T. parva* are shown in tabular format.

We compared the chromosomal location of each *rab* gene and the level of transcription and were able to rule out that chromosomal context influences transcription levels (Fig. 1). The 9 different *rab* genes are distributed over the 4 *Theileria* chromosomes with no obvious clustering. A similar genome-wide distribution of *rab* genes has been described for *Plasmodia* [21,22]. Given the high level of synteny between *T. annulata* and *T. parva* genomes each *rab* gene was positionally conserved located in a similar position in the two species. This is shown schematically for chromosome 2 (Fig. 1).

## 4 Transcription profiling of *P. falciparum rab* genes derived from microarray analysis

**Fig. 2:**
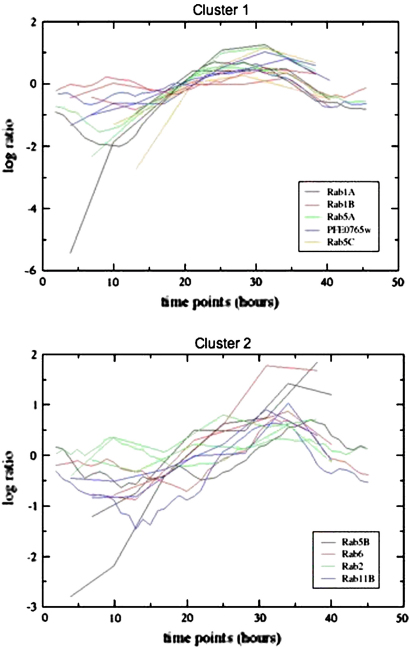
Expression profiles of individual *P. falciparum rab* genes in asexual life stages of the parasite. The genes *rab1a*, *rab1b*, *rab5a*, *rab5c*, and PFE0765w (*vps34*) are found in cluster 1, while *rab5b*, *rab6*, *rab2*, and *rab11b* are found in cluster 2. Expression profiles for each gene are colour coded as indicated in the figure. The *x*-axis shows the hours post-infection of a red blood cell by the parasite, and the *y*-axis shows the log(2) ratio of expression values for each normalised *rab* gene. For details of the microarray data see Supplementary Fig. S1.

There have been several published studies investigating genome-wide transcription of *P. falciparum* at different life cycle stages and at different points in intra-erythrocyte development. From these analyses has arisen the notion that genes with similar transcription profiles might be involved in similar biological processes [3,4]. With this in mind we wondered if *rabs* coding for similar processes like the early steps in endocytosis (Rab5A, 5B, 5C) might have similar profiles to a known Rab5 effector protein such as Vsp34 [23]. Data from a second array [14,24] profiled the transcriptome of *P. falciparum* at various points during the erythrocytic cycle of the parasite (early and late ring, early and late trophozoite, early and late schizont, free merozoite). The normalised data from both platforms were used separately for unsupervised non-hierarchical clustering (ArrayMiner, Optimal Design) and careful examination of the clusters revealed that the transcripts for *rab* genes fall into three groups. The *rab1a* and *rab1b*, *rab5a*, *rab5c* and *vps34* (PFE0765w) transcripts can be found in the same cluster and seem to peak at the trophozoite–late trophozoite stage (Fig. 2, top: cluster 1). In contrast, the *rab2*, *rab5b*, *rab6*, and *rab11b* transcripts fall into another cluster that seems to peak at late schizont stage (Fig. 2, bottom: cluster 2). The 3 other *rab* genes (*rab7*, *rab11a* and *rab18*) do not appear to share any specific expression profile and hence fail to fall in any cluster.

Transcripts from the Rab5 effector *vps34* (PFE0765w) are found in the same cluster as *rab5a* and *rab5c*, and not in the *rab5b* cluster. This could be taken as an indication that (1) Rab5B is not involved in the same biological process (endocytosis) as Rab5A and Rab5C and (2) Vps34 is a potential effector of Rab5A and/or Rab5C, but not Rab5B. We have previously pointed out that the presence of a 30 amino acid insertion in the effector domain of Rab5A should confer on its interactions with novel effectors [8]. This logic would favour Vps34 as a Rab5C effector. The dissociation of expression profiles of *rab5a* and *rab5c* from *rab5b* may be related to the observation that Rab5B appears to be a non-functional Rab, lacking the C-terminal prenylation motif necessary for its attachment to the vesicle membrane [8]. In spite of the absence of any prenylation motif, *rab5b* is transcribed and the coding sequence free of stop codons, implying that it plays some unusual regulatory function. As a non-vesicle associated Rab it is unlikely that Rab5B participates in endocytosis and therefore, perhaps not surprising that its expression profile does not cluster with *rab5a* and *rab5c*. Just what is the commonality between *rab5b*, *rab2*, *rab6* and *rab11b* suggested by their expression profiles is difficult to ascertain. Why *rab7*, *rab11a* and *rab18* fail to fall into any cluster is a mystery, especially given that Rab7, like Rab5 is normally a regulator of endocytosis.

## 5 The presence of specific binding motifs in the promoters of *rab* genes belonging to expression profiles of clusters 1 and 2

Even though *P. falciparum* undergoes many developmental changes with large variations in gene expression it encodes relatively few recognisable transcriptional regulators [17]. Recently, using a bioinformatic approach that integrated sequence conservation among species and correlations in mRNA expression (microarray data), 12 novel putative regulatory binding motifs were identified [16]. This analysis proposed a model that suggested that *Plasmodium* might use combinatorial binding of different protein factors to these motifs in the 5′-upstream regions (“promoters”) of genes to regulate transcription.

**Fig. 3:**
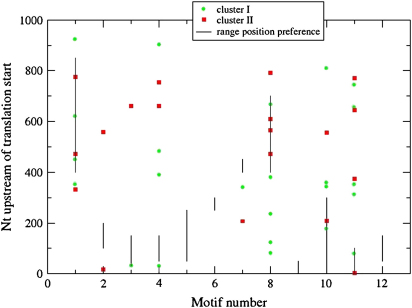
The presence and position of motifs [16] in 1 kb 5′ to the initiation codons of the *P. falciparum rab* genes is given for cluster 1 (green) and cluster 2 (red). Some motifs are present more than once and motifs 5, 6, 9 and 12 were not found in any *rab* upstream region.

We searched for the presence of these novel motifs in 1 kb 5′ to the initiation codons of the *P. falciparum rab* genes to see if members of a given cluster had a specific association with motifs that might explain their expression profiles (Fig. 3). Both the presence and the position of the motifs are noted in green (cluster 1) and red (cluster 2). Some motifs are present more than once and motifs 5, 6, 9 and 12 [16] were not found in any *rab* upstream region. There appears to be no discernable association of a particular combination of motifs that is cluster specific (Table 3).

## 6 Phylogenetic analysis indicated a core set of essential Rabs in Apicomplexa

In order to gain insights into what might constitute the minimal number of Rabs making up a core set for Apicomplexa we first established by repeated reciprocal BLAST analyses using *P. falciparum* Rabs as a query that *T. annulata* and *T. parva* code for a total of just 9 Rabs (see Table 1). The closely related *Babesia* has a similar number of 9 Rabs. Unlike *Plasmodia* parasites neither *Theileria*, nor *Babesia* encode Rab5B and Rab18. As stated, even though transcribed in *P. falciparum* Rab5B appears to be a non-functional Rab lacking a prenylation motif as its C-terminus [8]. Thus, its maintenance in both *P. falciparum* and *P. berghei* argues that Rab5B might play some novel regulatory function [11]. Importantly, *Theileria* and *Babesia* parasites differ from *Plasmodia* in that within host cells they do not reside inside a parasitophorous vacuole and the observation that they do not code for Rab18 may be related to the absence of the parasitophorous vacuole membrane, as a target membrane for vesicular transport.

**Fig. 4:**
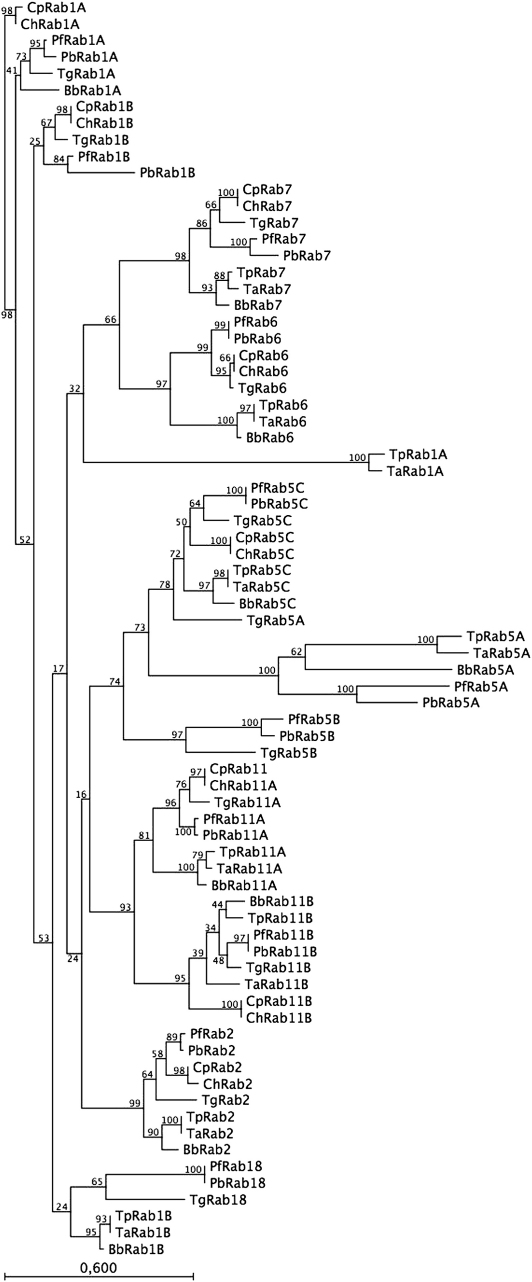
An unrooted neighbor-joining (NJ) tree showing the evolutionary relationships of amino acid sequences between the PM1 and PM3 domains [8] of Rab proteins from *Plasmodium falciparum* (Pf), *Plasmodium berghei* (Pb), *Theileria parva* (Tp), *Theleria annulata* (Ta), *Babesia bovis* (Bb), *Cryptosporidium parvum* (Cp), *Cryptosporidium hominis* (Ch), and *Toxoplasma gondii* (Tg). Bootstrap values are indicated on the branches. For a list of Rab sequences see Supplementary Fig. S2.

We choose two other Apicomplexa, *Toxoplasma* and *Cryptosporidia* and asked if these related parasites posses Rab5B and Rab18 (Fig. 4). *T. gondii* was chosen, as like *Plasmodia* it resides within a parasitophorous vacuole and *C. parvum* and *C. hominis*, as they lack an apicoplast [1,25]. As we have previously remarked, similar Rabs from different *Plasmodia* parasites form clads with yeast Rabs (Ypts) suggesting potentially similar function [8,11]. Relevant here, is that the Rab5B clad only contains *P. falciparum*, *P. berghei* and *T. gondii* (Fig. 4). A Rab5B orthologue can also be detected in *N. caninum* (data not shown) arguing that any putative novel regulatory function performed by this unusual Rab might be common to *Plasmodia* and *Toxoplasma*/*Neospora* that reside within a parasitophorous vacuole.

In plants Rab18 is induced by stress such as drought and its induction is thought to mobilise lipid food reserves [26]. In eukaryotes Rab18 is also associated with lipid droplets (LDs), which have been traditionally been considered relatively inert storage organelles [27]. Overexpression of Rab18 induces association of LDs with membrane cisternae connected to the rough ER [28]. The presence of Rab18 only in *Plasmodia* and *Toxoplasma*/*Neospora* could be taken as an indicator that Rab18 might be involved in mobilising parasite lipid reserves that will be used in the formation of the parasitophorous vacuole.

## 7 Discussion

*Theileria* parasites have just 9 Rabs, unlike *Plasmodium*, they do not have Rab5B and Rab18. We compared the *Theileria* Rab family to those of *Babesia*, *Plasmodia*, *Toxoplasma* and *Cryptosporida* to determine whether 9 represented the “core” set of minimal Rabs common to Apicomplexa (Fig. 4). Rabs have divergent N- and C-termini and as a consequence correct annotation of full-length Rabs sequences is often lacking in the different Apicomplexa databases and therefore, only circa 48 amino acids between the first two (PM1 and PM3) GTP-binding motifs [8] were used to construct the tree presented in Fig. 4. This revealed that *Cryptosporidia* with just 8 Rabs defines the smallest set of Rabs, while *Toxoplasma* with 15 (and *Neospora*, not shown) have the largest. *Cryptosporidia* differ from *Theileria* in lacking Rab5A and have only a single early endosome-specific Rab (Rab5C).

**Fig. 5:**
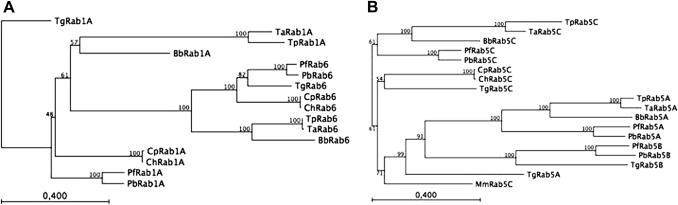
A) An unrooted neighbor-joining (NJ) tree showing the evolutionary relationships of full-length sequences of Rab1A, Rab6 and Rab11A from *Plasmodium falciparum* (Pf), *Plasmodium berghei* (Pb), *Theileria parva* (Tp), *Theleria annulata* (Ta), *Babesia bovis* (Bb), *Cryptosporidium parvum* (Cp), *Cryptosporidium hominis* (Ch), and *Toxoplasma gondii* (Tg). (B) A rooted neighbor-joining (NJ) tree showing the evolutionary relationships of full-length sequences of Apicomplexa Rab5A, Rab5B and Rab5C. The tree was rooted on Rab5C (GeneID: 19345) from *Mus musculus* (Mm). Bootstrap values are indicated on the branches. For full-length Rab1A sequences see Supplementary Fig. S3 and for full-length Rab5 sequences see Supplementary Fig. S4.

There are some notable features of the tree in Fig. 4. First, using just these “effector domain” amino acids between the PM1 and PM3 motifs [8], Rab1A from *T. parva* and *T. annulata* was found to cluster with Rab6, rather with Rab1A of the other Apicomplexa. Consequently, we repeated the phylogenic analysis with full-length Rab sequences for Rab1A, Rab6 and Rab11A for all 7 Apicomplexa (Fig. 5A). This shows that Rab1A from the two *Theileria* now clusters with the other Rab1As and particularly with *Babesia*, as expected. Due to the unusually long N-terminus of Rab1A from *Toxoplasma* (see Supplementary Fig. S4) it now appears as an outlier in this analysis and underscores why we used only amino acids between the PM1 and PM3 motifs in Fig. 5. Taken together, it would appear that *Theileria* posses an unusual Rab1A with a divergent, Rab6-like effector domain compared to Rab1As of the 6 other Apicomplexa.

The second notable feature is the Rab5A clad with the long branch-lengths arguing that Rab5A is evolving faster than other Rabs of Apicomplexa (Fig. 4). You can note that this clad lacks Rab5A from *Toxoplasma* that appears in the Rab5C clad together with the *Toxoplasma* Rab5C orthologue. Individual BLAST analysis indicates that TgRab5A is almost similar to PfRab5A and PfRab5C. Taken as an ensemble the Rab5 clad (Rab 5A, 5B, 5C) is highly diverse and this divergence may be a reflection of adaptation of individual parasites to their specific intracellular environment (Fig. 5B). For example, Rab5A of *Plasmodia* is most unusual in having a 30 amino acid insertion in its effector domain and we have argued that this insertion could mediate interactions with novel Rab5 effector proteins [8,11]. This insertion is not observed in Rab5A of *Theileria*, *Babesia* and *Toxoplasma* strengthening the argument that any putative novel PfRab5A effectors are *Plasmodia*-specific. *Plasmodia* differ from *Theileria*, *Babesia* and *Toxoplasma* in that they import haemoglobin from infected erythrocytes (*Babesia* has no parasitophorous vacuole, nor is it known to degrade haemoglobin) and it is interesting to speculate that these putative novel *Plasmodia* Rab5A effectors might be involved in regulating uptake of haemoglobin.

The lack of Rab5B in *Theileria* might not be considered surprising given that this Rab seems to lack C-terminal geranyl-geranylation motifs [8]. Even though not attached to a vesicle, Rab5B is nonetheless expressed and probably binds some effector molecules and therefore, could play a regulatory function through sequestering these effectors into incompetent complexes. The maintenance of Rab5B in *Toxoplasma* that also lacks recognisable C-terminal geranyl-geranylation motifs argues that here also it is playing some unusual, perhaps similar, regulatory role. In the same vain, only *Plasmodia* and *Toxoplasma*/*Neospora* appear to have Rab18 (Fig. 5). We mentioned that Rab18 might be involved in mobilising parasite lipid reserves required for parasitophorous vacuole formation, but if so, it would argue that their parasitophorous vacuole differs from that of *Crypstosporida* and this indeed is the case [9].

The combined transcriptional profiling analysis of *rab* genes in *Theileria* (EST and MPSS profiling) and *Plasmodia* (microarray) concur and indicate that all Apicomplexa *rab* genes are transcribed all the time. MPSS scores in *T. parva* indicate that amounts of transcript for an individual *rab*, however, are generally low (10× lower than *ran*, see Table 2), but can vary for a given *rab* gene; for example, *rab2* transcripts in *T. parva* are 60× more abundant than those for *rab1a*. To try and understand how parasites are controlling the levels of *rab* transcription we exploited the published *P. falciparum* microarray data and tried to correlate expression levels with the presence of a combination of specific motifs [16] in the 5′-regions 1 kb upstream of each *P. falciparum rab* gene (Figs. 2 and 3). We also asked if *rabs* with similar transcription profiles might have similar function, as suggested by their presence in the same cluster on the phylogenetic tree (Fig. 4). We could observe no obvious relationship between, expression profiles, a particular combination of promoter motifs and putative Rab function (Table 3).

There are a number of interesting concepts that stem from our analysis of *rab* gene expression, whether estimated by MPSS in *T. parva*, or microarray analysis in *P. falciparum*. Clearly, other factors such as mRNA stability may come into play and explain why some transcripts accumulate, while others do not. However, taken together, our analysis suggests that the structure of the “promoter” regions of *rab* genes may determine accessibility of the transcriptional machinery to the upstream regions of genes, *i.e.* the structure determines the level of chromatinisation and has a strong influence on transcription in Apicomplexan parasites. Low general levels of chromatinisation may explain why all (*rab*) genes are transcribed all the time and genes with readily accessible “promoters” are preferentially transcribed for the first few hours post-invasion.

Rabs in Apicomplexa thus provide insights not only into novel aspects of how intracellular protozoan parasites might regulate vesicular traffic, but also into (*rab*) gene transcription that may be controlled.

## Figures and Tables

**Table 1:** For the 11 different *P. falciparum* Rabs is given the PlasmoDB identification (ID) and the corresponding accession number

*P. falciparum*	ID	Accession numbers	*T. parva*	ID	*T. annulata*	ID	Schizont EST	Piro EST
PfRab1A	PFE0690	AF201953	TpRab1A	TP04_0644	TaRab1A	TA10730	–	–
PfRab1B	PFE0625w	AJ409198	TpRab1B	Tp03_0775	TaRab1B	TA18180	+	+
PfRab2	PFL1500w	AJ308736	TpRab2	TP01_0877	TaRab2	TA06165	+	–
PfRab5A	PFB0500c	AAC71889	TpRab5A	TP04_0575	TaRab5A	TA09125	–	–
PfRab5B	MAL13P1.51	AJ422110	–		–			
PfRab5C	PFA0335w	AJ420321	TpRab5C	TP01_0639	TaRab5C	TA03030	+	–
PfRab6	PF11_0461	X92977	TpRab6	TP02_0799	TaRab6	TA15190	–	+
PfRab7	PF10155c	AJ290938	TpRab7	TP03_0666	TaRab7	TA17640	+	–
PfRab11A	PF130119	X93161	TpRab11A	TP01_1204	TaRab11A	TA09700	+	–
PfRab11B	MAL03P1.205	AJ879563	TpRab11B	TP02_0559	TaRab11B	TA13860	+	–
PfRab18	PF08_0110	AJ438271	–		–			

The *T. parva* TIGR Database and *T. annulata* GeneDB identification numbers are given for each of the 9 different *Theileria* Rabs. When detected the presence of schizont and prioplast ESTs are indicated for each *Theileria* Rab.

**Table 2:** The massively parallel signature sequencing (MPSS) values, presence of sense and antisense transcripts and chromosomal locations are shown for each of the 9 *T. parva rabs*

Name	TPM	Orientation	Chromosome	Locus	Common name
*Tprab5C*	5	Sense	1	TP01_0197	GTP-binding protein, putative
53	Sense	1	TP01_0639	GTP-binding protein Rab5, putative
*Tpran*	1178	Sense	1	TP01_0757	GTP-binding nuclear protein ran, putative
*Tprab2*	261	Sense	1	TP01_0877	GTP-binding protein Rab2, putative
*Tprab11A*	143	Sense	1	TP01_1204	GTP-binding protein Rab11, putative
*Tprab11A*	26	Anti-sense	1	TP01_1204	GTP-binding protein Rab11, putative
229	Sense	2	TP02_0248	GTP-binding nuclear protein 1, putative
*Tprab6*	22	Sense	2	TP02_0799	GTP-binding protein Rab6, putative
*Tprab11B*	none		2	TP02_0559	GTP-binding protein Rab11b, putative
77	Sense	3	TP03_0502	GTP-binding protein, putative
429	Sense	3	TP03_0578	GTP-binding protein, putative
539	Anti-sense	3	TP03_0578	GTP-binding protein, putative
*Tprab1B*	none		3	TP03_0775	GTP-binding protein Rab1b, putative
*Tprab7*	82	Sense	3	TP03_0666	GTP-binding protein Rab7, putative
*Tprab5A*	175	Sense	4	TP04_0575	GTP-binding protein, Rab5A putative
*Tprab1A*	4	Sense	4	TP04_0644	GTP-binding protein, Rab1A putative

The locus position for each *rab* is indicated by the corresponding TIGR Database gene ID number. Included for comparison are the MPSS scores for genes coding for number of putative GTP-binding proteins.

**Table 3:** Presence of regulatory elements in upstream regions of *rab* genes and effectors

		1	2	3	4	5	6	7	8	9	10	11	12	Max-exp
PFE0690c	Rab1A				17			20	12		15	15		24
PFB0500c	Rab5A	12		16	17				12			15		25
PFA0335w	Rab5C	12			17				12		16	14		25
PFE0765w	Vps34	12			13				12		16	14		30
PFE0625w	Rab1B	12							12		14	14		30
PF11_0461	Rab6	12	20					20	12		19	15		32
MAL13P1.205	Rab11B				14				12			14		32
PF13_0057	Rab5B	12		19	16				12		16	14		37
PFL1500w	Rab2	12	21						12			15		10
PF08_0110	Rab18	12			21				12		14	14		9
PF13_0119	Rab11A	12			15				12			14		25
PFI0155c	Rab7				14	18		15	11			15		30

Genes are ordered into cluster 1, cluster 2 and the left over cluster. Within the clusters the genes are ordered to the time of maximum level of mRNA expression for that gene. For each motif [16, p. 19], the highest score of the upstream region with that motif is given, together with the time after infection when that gene has maximum mRNA expression. For motif 4, a higher score seems to indicate earlier expression. NB: *Rab5c* is expressed shortly before the Rab5 effector gene PFE0765w (*vps34*). If you take into account that the expression arrays of 10 and 45 h after infection PF14_0689 (Yip1p) is also close to *rab2*, but it would be synthesized first. Rab7 and MAL6P1.286 (PfMyoE) are both in the left over cluster and expressed shortly after each other. This is not true for PF10_0046 (Rabring 7).
