# Supplementary material for: Comparative genomics of the Rab protein family in Apicomplexan parasites
Source: Microbes Infect. 2008 Apr;10(5):462–70. doi: 10.1016/j.micinf.2008.01.017 (PMC3317772; doi:10.1016/j.micinf.2008.01.017)
Supplement: Supplementary file 1 [file mmc1.doc]

The *P. falciparum* scrMalaria custom array {Le Roch, 2002 #26} {Le Roch, 2003 #27} contains 506,944 probes, each consisting of 25 nucleotides and only perfect match probe sequences were placed on the array. Thus, there are 5340 probesets on the array, each corresponding to one predicted gene. The number of probes per gene ranges from a single probe up to hundreds for genes with very long sequences. The DNA microarray exploited in this study consists of 7,462 individual 70mer oligonucleotides representing 4,488 OFRs, where 990 are represented by more than one oligonucleotide {Bozdech, 2003 #17; Bozdech, 2003 #16}.

Le Roch and colleges {Le Roch, 2003 #27} performed a study in which they profiled the transcriptome of *P. falciparum* at various points during the life cycle, using the custom chip described above. This data set included samples for the gametocyte and sporozoite stages, as well as seven different time points during the erythrocytic cycle of the parasite (early and late ring, early and late trophozoite, early and late schizont, free merozoite). Although two synchronization methods were used to treat the erythrocytic cycle samples, in our analysis present here, we only used the sorbitol synchronized data set and downloaded the raw files from <http://carrier.gnf.org/publications/CellCycle/>. Bozdech et al. (2003) performed a similar study in which they profiled the intra-erythrocytic transcriptome of *P. falciparum* by taking samples all along the 48h life cycle of the parasite. The raw data set was downloaded from <http://malaria.ucsf.edu/comparison/comp_SupplementalData.php>.
