# Supplementary material for: Comparative genomics of the Rab protein family in Apicomplexan parasites
Source: Microbes Infect. 2008 Apr;10(5):462–70. doi: 10.1016/j.micinf.2008.01.017 (PMC3317772; doi:10.1016/j.micinf.2008.01.017)
Supplement: Supplementary file 2 [file mmc2.doc]

>ChRab5C

SCLVVRFSKDEFHEYQEPTIGAAFMTQSVNVDDDYTVKFEIWDTAGQE

>CpRab5C

SCLVVRFSKDEFHEYQEPTIGAAFMTQSVNVDDDYTVKFEIWDTAGQE

>TgRab5C

SCLVVRFAKDEFYEYQESTIGAAFMTQSVNLGSCIVKFEIWDTAGQE

>PbRab5C

SCIVVRFAKNEFYEYQESTIGAAFMTQLIDIGECTIKFEIWDTAGQE

>PfRab5C

SCIVVRFAKNEFYEYQESTIGAAFMTQLIDIGECTIKFEIWDTAGQE

>TaRab5C

SCLVGRFVKNTFMEFQESTIGAAFMTQTVKLDDCNVKFEIWDTAGQE

>TpRab5C

SCLVGRFVKNTFMEFQESTIGAAFMTQTVKLDDCNVKFEIWDTAGQE

>BbRab5C

SSLVGRFVKNTFLEFQESTIGAAFMTQSVSLDDCTVKFEIWDTAGQE

>TgRab5A

SSLVVRFVKNTFSDTMETTIGAAFFTQALQVDGRTVKFEIWDTAGQE

>PbRab5A

SSIVLRLTKDTFHDNTNTTIGASFCTYVVNINELKTNNNNGDNRSNNNNNLTFND

ENSESLYNIKFDIWDTAGQE

>PfRab5A

SSIVLRLTKDTFHENTNTTIGASFCTYVVNLNDINIKNNSNNEKNNNINSINDDNNVIITNQ

HNNYNENLCNIKFDIWDTAGQE

>TaRab5A

SSFAVRLTKGEFSDNTNSTIGAAFFTYTVHNDGSDVQKRLNLNTDGKFDDKLVDGVSQSEVRSSS

IASNYDENFTIKFDIWDTAGQE

>TpRab5A

SSFAVRLTKGEFSDNTNSTIGAAFFTYTVHNDYSDPQKRLNINSDGKFTDKLSDGPSQSQQSS

IISHYDENITIKFDIWDTAGQE

>BbRab5A

SSFVLRLTRGEFADSTTSTIGAAFFMHTVRCSPPAAKAVTESAAPKDGSSTRGIQTPTS

QDDASGAQVTVKFDIWDTAGQE

>PbRab5B

SSIALYLCHGRFSDSHQVTIGAAFLHHTIHLKNGETMKLHIWDTGGQE

>PfRab5B

SSIALYLCHGRFSEKHQVTIGAAFLHHNIELKNGATMKLHIWDTGGQE

>TgRab5B

SSLALRFCRGRFPQYHEVTIGAAFLQQTIRVGDDGSQLKLYIWDTGGQE

>ChRab6

TSIITRFMYDTFDNNYQATIGIDFLSKTLYLEDRTIRLQLWDTAGQE

>CpRab6

TSIITRFMYDTFDNNYQATIGIDFLSKTLYLEDRTIRLQLWDTAGQE

>PbRab6

TSIITRFMYDTFDNNYQSTIGIDFLSKTLYLDEGPVRLQLWDTAGQE

>PfRab6

TSIITRFMYDTFDNNYQSTIGIDFLSKTLYLDEGPVRLQLWDTAGQE

>TgRab6

TSIITRFMYDTFDNNYQATIGIDFLSKTLYLEDRTVRLQLWDTAGQE

>TaRab6

TSIVTRFVYDHYIPAYAATIGIDFLSKVVTVNQKTMRLQLWDTAGQE

>TpRab6

TSIVTRFVYDHYIPAYAATIGIDFLSKVVTVNQKTMRLQLWDTAGQE

>BbRab6

TSIVTRFVYDHFVPAYAATIGIDFLSKVVTVNGKTMRLQLWDTAGQE

>ChRab7

TSLMNQYVNSKFSTQYKATIGADFLTKDIVIDNKLVTLQIWDTAGQE

>CpRab7

TSLMNQYVNSKFSTQYKATIGADFLTKDIVIDNKLVTLQIWDTAGQE

>TgRab7

TSLMNQYVNKKFSNQYKATIGADFLTKDVVIDDKEVTVQIWDTAGQE

>PbRab7

LMNQYVNKKFTNQYKATIGADFLTKETVVDNEQLTMQVIWDTAGQE

>PfRab7

TSLMNQYVNKKFTNQYKATIGADFLTKETIVDNEQITMQIWDTAGQE

>TaRab7

TSLMNQFINKRFTNQYRATIGADFLTMEMTVDDKEVTLQIWDTAGQE

>TpRab7

TSLMNQFINKRFTNQYRATIGADFLTMGITVDDKEVTLQIWDTAGQE

>BbRab7

TSLMNQFINKRFTNQYRATIGADFSTQEVTVDDKVVTLQIWDTAGQE

>ChRab11A

SNLLSRFTRDEFNLESKSTIGVEFATKSIITEGKVIKAQIWDTAGQE

>CpRab11

SNLLSRFTRDEFNLESKSTIGVEFATKSIITEGKVIKAQIWDTAGQE

>PbRab11A

SNLLSRFTRDEFNLESKSTIGVEFATKSIQLKNDKIIKAQIWDTAGQE

>PfRab11A

SNLLSRFTRDEFNLESKSTIGVEFATKSIQLKNNKIIKAQIWDTAGQE

>TgRab11A

SNMLSRFTRDEFNLESKSTIGVEFATKSVYLDEGKVIKAQIWDTAGQE

>TaRab11A

SNLLDRFVKGNFKLDSKSTIGVEFATKNVNLKNGKIAKAQIWDTAGQE

>TpRab11A

SNLLDRFVKGNFKLDSKSTIGVEFATKNVNLRNGKVAKAQIWDTAGQE

>BbRab11A

SNLLDRFVKGNFKLDSKSTIGVEFATKTVTLNNGKIAKAQIWDTAGQE

>ChRab11B

THLLSRYTRDALPKTPQPTIGVEFATRTVPLSIGGTVKAQIWDTAGQE

>CpRab11B

THLLSRYTRDALPKTPQPTIGVEFATRTVPLSIGGTVKAQIWDTAGQE

>TgRab11B

THLLSRYIRGTLPKSPKATIGVEFATRTVPLAVGGTVKAQIWDTAGQE

>PbRab11B

THLLSRYIRGSLPSVAKATIGVEFATRTIPLAVGGTVKAQIWDTAGQE

>PfRab11B

THLLSRYIRGSLPSVAKATIGVEFATRTIPLAVGGTVKAQIWDTAGQE

>TaRab11B

SHLLCRYIKGNLPIQSKATIGVEFATKTVPLASGGSIKAQIWDTAGQE

>TpRab11B

SHLLCRYIRGDLPVQAKATIGVEFATRTVPLASGGSVKAQIWDTAGQE

>BbRab11B

SHLLSHYIRGTLPKQAKATIGVEFATRTVPLASGGTVKAQIWDTAGQE

>ChRab2

SCLLLQFTDRRFRVDHDLTIGVEFGARIINLDAKKIKLQIWDAGQE

>CpRab2

SCLLLQFTDRRFRVDHDLTIGVEFGARIINLDAKKIKLQIWDTAGQE

>PbRab2

SCLLLQFTDKRFRADHDLTIGVEFGARLINLDNKQIKLQIWDTAGQE

>PfRab2

SCLLLQFTDKRFRADHDLTIGVEFGARLVNLDNKQIKLQIWDTAGQE

>TgRab2

SCLLLQFTDKRFRTDHDLTIGVEFGARLVSIAGRQVKLQIWDTAGQE

>TaRab2

SCLLLRFTDKRFKPDHDLTIGVEFGTRFITVQGEQIKLQIWDTAGQE

>TpRab2

SCLLLRFTDKRFKPDHDLTIGVEFGTRFITVQGEQIKLQIWDTAGQE

>BbRab2

SCLLLRFTDKRFKPDHDLTIGVEFGTRLINVKGDTIKLQIWDTAGQE

>PbRab18

SSILCRYSDNQFEEKVLSTIGIDFKVKYLKIDNKTIKVGIWDTAGQE

>PfRab18

SSILCRYSDNQFEEKVLSTIGIDFKVKYLKIDNKTIKVGIWDTAGQE

>TgRab18

SSILLRFAEDVFNEKQLSTIGVDFKVKAMTVGDMRLKLAIWDTAGQE

>ChRab1B

SCLLLRFADDTYTDSYISTIGVDFKIRTISLENKTVKLQIWDTAGQE

>CpRab1B

SCLLLRFADDTYTDSYISTIGVDFKIRTISLENKTVKLQIWDTAGQE

>TgRab1B

SCLLLRFADDTYTESYISTIGVDFKIRTIDLDGKTVKLQIWDTAGQE

>PbRab1B

SCLLLTYCCNQTLKIDDTYTDSYISTIGVDFKIKTIEIDDKIIKLQIWDTAGQE

>PfRab1B

SCLLLRFADDTYTDSYISTIGVDFKIKTIEIEDKIIKLQIWDTAGQE

>TaRab1B

SSLLLRFADNTYSESYMSTIGVDFKIKTVKIDNTTIKLQIWDTAGQE

>TpRab1B

SSLLLRFADNTYSESYMSTIGVDFKIKTVKIDNTTIKLQIWDTAGQE

>BbRab1B

SSLLLRFADDTYSESYMSTIGVDFKIKTVKIDNVTIKLQIWDTAGQE

>ChRab1A

SCLLLRFADDSFTDSYITTIGVDFRFRTIKIDDKIIKLQIWDTAGQE

>CpRab1A

SCLLLRFAQDDSFTDSYITTIGVDFRFRTIKIDDKIIKLQIWDTAGQE

>PbRab1A

SCILLRFSDDHFTESYITTIGVDFRFRTLKVDDKVVKLQIWDTAGQE

>PfRab1A

SCILLRFSDDHFTESYITTIGVDFRFRTIKVDDKIVKLQIWDTAGQE

>TgRab1A

SCLLLRFSDDAFTESYITTIGVDFRFRTINVDNEIVKLQIWDTAGQE

>BbRab1A

SCVLLRFADDTFTDSYITTIGVDFRFRTIEVEGRRVKLQIWDTAGQE

>TaRab1A

SCLLLRFTDKRFKPDHDLTIGVEFGTRFITVQGEQIKLQIWDTAGQE

>TpRab1A

SSLLLRFADNTYSESYMSTIGVDFKIKTVKIDNTTIKLQIWDTAGQE

>NcRab7

TSLMNQYVNKKFTNQYKATRSAVGADFLTKDVVIDDKEVTVQIWDTAGA

>NcRab5A

VVRFVKNTFSDTMETTIGAAFFTQALQVDGRTVKFEIWDTAGQE

>NcRab1A

VLMRFVDQYSAFHVSTIGVDFKSVLTMVKGKVCTLQLWDTAGQE

>NcRabX

LLSRFAQGRFVDHRTTLGVDFETRTLDIDGKRVKIQLWDTAGHE

>NcRab6

VFFMYDTFDNNYQQATIGIDFLSKTLYLEDRTVRLQLWDTAGQE

>NcRab18

LLISEDVFNEKQLSTIGGVDFKVKAITVGDARLKLAIWDTAGQE

>NcRab1B

ILLRFDDTYTESYISTIGVDFKIRTIDLDGKTVKLQIWDTAGQE

>NcRab5B

ALRFCRGRFPQYHEVTIGAAFLQQTVKVANDQLKLYIWDTGGQE

>NcRab5C

VVRFAKDEFYEYQESTIGAAFMTQSVDLGSCIVKFEIWDTAGQE

>NcRab11A

RRFTRDEFNLESKSTIGVEFATKSVYLDEGKVIKAQIWDTAGQE

>NcRab2

LLQFTDKRFRTDHDLTIGVEFGARLISIAGRQVKLQIWDTAGQE
