# Supplementary material for: Comparative genomics of the Rab protein family in Apicomplexan parasites
Source: Microbes Infect. 2008 Apr;10(5):462–70. doi: 10.1016/j.micinf.2008.01.017 (PMC3317772; doi:10.1016/j.micinf.2008.01.017)
Supplement: Supplementary file 3 [file mmc3.doc]

>TpRab1A

MCSFPGSFSPLSLVIISSNSFSSVIVALESPVCCSDLTLFVKDKRVKLQIWDTAGQERFRTITSTYYRGADGIIMVYDITDKSSFEHINDWLTEVNKYSSEDTCKLLLGNKSDMEDSRDVSATDVQRLSEVIEVPAMDVSAKTGHNIDKDVFQAFYSITEKLVSLKMESDHDVTDLRTISLQNTYNGTF

>TaRab1A

MCFFPTPFSPFSLVIISSNSFSSAIVALENLAYCSDLLTIPLRIVTSLLSVWILTLFVKDKRVKLQIWDTAGQERFRTITSTYYRGADGIIMVYDITDKSSFEHINDWLSEVNKYSSEDTCKLLLGNKCDMEDNRDVSLTDVQRLSEVIEVPAMDVSAKTGHNIDKAFYSITEKLVSLKMESDHDVVDLRTISLQNTYNGIFCFNLLL

>BbRab1A

MVSRAAKDYDHLFKLVLIGDSGVGKSCVLLRFADDTFTDSYITTIGVDFRFRTIEVEGRRVKLQIWDTAGQERFRTITSAYYRGADAIIIVFDITDKLSFENVPSWLQEVEKFAPDGIHKLLIGNKSDQAQARDVDPSEIQEFSELHSTPYVEISAKSGSNVEEAFVSVARRLVIDRHGNDPSSNQSIPQPISLNDRVKSVVESIYGGNKLCCS

>ChRab1A

MSAVRQKEDFLFKLVLIGDSGVGKSCLLLRFADDSFTDSYITTIGVDFRFRTIKIDDKIIKLQIWDTAGQERFRTITSAYYRGADGVVLVYDTTSTSSFDHIDEWVTEVNRYTTDSTKILIGNKCDLTSQKMVDFATGQKKAQELQVDFMESSAKNSTNVEECFVNIARKLLEKKLKNGETSSKPINSQNIYLNQHGDGGLISGVLSSCCK

>CpRab1A

MSAVRQKEYDFLFKLVLIGDSGVGKSCLLLRFAQDDSFTDSYITTIGVDFRFRTIKIDDKIIKLQIWDTAGQERFRTITSAYYRGADGVVLVYDTTSTSSFDHIDEWVTEVNRYTTDSTKILIGNKCDLTSQKMVDFATGQKKAQELQVDFMESSAKNSTNVEECFVNIARKLLEKKLKNGETSSKPINSQNIYLNQHGDGGLISGVLSSCCK

>PbRab1A

MNENRSRDYDYLYKIILIGDSGVGKSCILLRFSDDHFTESYITTIGVDFRFRTLKVDDKVVKLQIWDTAGQERFRTITSAYYRGADGIIIIYDTTDRNSFLHINDWMAEINKYTNDDTCKLLVGNKFDCKDEIEVPTAEGESKAKELNIP

FIETSAKDALNVELAFTMITQELIKKKKKKNINSVNNQTKVNLFSDDRDQSSLCSC

>PfRab1A

MTENRSRDYDYLYKIILIGDSGVGKSCILLRFSDDHFTESYITTIGVDFRFRTIKVDDKIVKLQIWDTAGQERFRTITSAYYRGADGIIIIYDTTDRNSFLHINDWMNEINKYTNEDTCKLLVGNKADCKDDIEITTMEGQNKAKELNISFIETSAKDATNVELAFTMITQELIKKKKKKNFTSLKNNHAKLKLSTHDNSPQSFCSC

>TgRab1A

MRLWWLARASLRPTRGNWPLDRVQLCARIPLDHLFKLVLIGDSGVGKSCLLLRFSDDAFTESYITTIGVDFRFRTINVDNEIVKLQIWDTAGQERFRTITSAYYRGADGIVLVYDVTDRESFLHVDEWLAEVNRYANENTCKILVGNKCEKADDRQVSVEEGQRKAEELGISFIETSAKNAINVDEAFTVVARELIKMKQSAGAAAGRGVGPQGMRIASQPTGADNRAMAQRTGNCSC

>ChRab11A

MSSKDEHYDYLYKIVLIGDSGVGKSNLLSRFTRDEFNLESKSTIGVEFAT

KSIITEGKVIKAQIWDTAGQERYRAITSAYYRGAVGALLVYDISKRSSFE

NVERWLKELRDHADPNIVVLLVGNKSDLRNLRTVTQEEACAFSEREGMAC

MEASALNSSNVDEAFHRILSEIYTLRSERQLTANQHDLNKATLPLGTQGV

RVDPIRIDLNSKGSKKRSCC

>CpRab11A

MSSKDEHYDYLYKIVLIGDSGVGKSNLLSRFTRDEFNLESKSTIGVEFAT

KSIITEGKVIKAQIWDTAGQERYRAITSAYYRGAVGALLVYDISKRSSFE

NVERWLKELRDHADPNIVVLLVGNKSDLRNLRTVTQEEACAFSEREGMAC

MEASALNSSNVDEAFHRILSEIYTLRSERQLTANQHDLNKATLPLGTQGV

RVDPIRIDLNSKGSKKRSCC

>PbRab11A

MSMKEDYYDYLFKIVLIGDSGVGKSNLLSRFTRDEFNLESKSTIGVEFAT

KSIQLKNDKIIKAQIWDTAGQERYRAITSAYYRGAVGALLVYDITKKNTF

ENIEKWLKELRDNADSNIVILLVGNKSDLKHLRVINDNDATQFAKKEKLA

FIETSALEATNVELAFHQLLNEIYNVRQKKQATKSEDNVNIQPRG

>TgRab11A

MAAKDEYYDYLYKIVLIGDSGVGKSNMLSRFTRDEFNLESKSTIGVEFATKSVYLDEGKV

IKAQIWDTAGQERYRAITSAYYRGAVGALLVYDITKRQSFENVERWLKELRDHADPNIVI

LLVGNKSDLKHLRAVSVEEATKFANREHLAFIETSALDATNVEQAFHQILAEIYLLRQKK

QIEDNPQSTTQPGRGQKIHLDEERTDSQIRQSRRGCCSA

>PfRab11A

MAMKEDYYDYLFKIVLIGDSGVGKSNLLSRFTRDEFNLESKSTIGVEFAT

KSIQLKNNKIIKAQIWDTAGQERYRAITSAYYRGAVGALLVYDITKKNSF

ENIEKWLKELRDNADSNIVILLVGNKSDLKHLRVINDNDATQYAKKEKLA

FIETSALEATNVELAFHQLLNEIYNVRQKKQATKNDDNLSIQPRGKKINV

DDDNDKNETKKKNKCC

>TaRab11A

MAEQTYDYLFKIVLIGDSNVGKSNLLDRFVKGNFKLDSKSTIGVEFATKN

VNLKNGKIAKAQIWDTAGQERYRAITSAYYRGARGAIVVYDIASKQSFYN

VSRWLSELNEYGDPNMIIALVGNKSDLTHLREVTFEDGERYARSNNLLFF

ETSCLNNENIDTTFGELLNLICDNHEKFGETVTGSQVSKTITLTKPKKMK

KKLSKCC

>TpRab11A

MALDQNYDYLFKIVLIGDSNVGKSNLLDRFVKGNFKLDSKSTIGVEFATK

NVNLRNGKVAKAQIWDTAGQERYRAITSAYYRGARGAIVVYDIASKQSFY

NVSRWLSELNEYGDANMIIALVGNKSDLTHLREVTYEDGERYAKSNNLIF

FETSCLSNENIDTTFTELLNLICDNHEKFGDTSVTANGANVTKSLVSLAK

PKKMKKKMAKCC

>ChRab11B

MGSSPDEYDHLYKIILVGDATVGKTHLLSRYTRDALPKTPQPTIGVEFAT

RTVPLSIGGTVKAQIWDTAGQERYRAITRAHYRRSVGALLVYDITRKSSF

LNASKWLEDIKQNSEPDIVVMLVGNKLDLVEKDPSKREVPFDIAANFAQE

NNLFFSEASAVTRCNVKHIFEHLLQEVYNQKMKDNSSNLDMNGKFIKNC

>CpRab11B

MGSSPDEYDHLYKIILVGDATVGKTHLLSRYTRDALPKTPQPTIGVEFAT

RTVPLSIGGTVKAQIWDTAGQERYRAITRAHYRRSVGALLVYDITRKSSF

LNASKWLEDIKQNSEPDIVVMLVGNKLDLVEKDPSKREVPFDIAANFAQE

NNLFFSEASAVTRCNVKHIFEHLLQEVYNQKMKDNSSNLDMNGKFIKD

>PbRab11B

MSNEEYDHLYKIILVGDATVGKTHLLSRYIRGSLPSVAKATIGVEFATRT

IPLAVGGTVKAQIWDTAGQERYRSITSAHYRRSAGAILVYDITKKKSFLN

ISKWLEEIRQNSEKDIVIMLVGNKVDLAEEDETKRKVTYEQGASFAKENN

LFFSEASAVSKLNVKHIFENLLQIYNNRLKDSNSCSSTRSHETYEXIQIT

NAKNIIKLNDTNEKYNENNPNQMKCC

>PfRab11B

MSNEEYDHLYKIILVGDATVGKTHLLSRYIRGSLPSVAKATIGVEFATRT

IPLAVGGTVKAQIWDTAGQERYRSITSAHYRRSAGAILVYDITKKKTFLS

ISKWLEEIRQNADKDIVIMLVGNKVDLTEEDETKRKVTYEQGANFARENN

LFFAEASAVSKLNVKHIFENLLQEIYNNRLKNNNRSFSNRSVATCESAIQ

LTKARSVIKLNEVYDNQSEDNNMNKVKCC

>TgRab11B

MAGGSEDYDHLYKVILVGDATVGKTHLLSRYIRGTLPKSPKATIGVEFATRTVPLAVGGT

VKAQIWDTAGQERYRSITSAHYRRAVGALLVYDVTRKSTFLNASKWLEELRQNSEPDIVI

MMVGNKLDLVEKDPTARDVPYELAAKFAQANGLYFSEASAVTAFNVKHIFEHLLQEIYNH

RTQGEEFSGRANGADAYRDAQALGGVRLAANANMYGRNPQNLSCCG

>TaRab11B

BMAEEYEHVYKIILLGDATVGKSHLLCRYIKGNLPIQSKATIGVEFATKT

VPLASGGSIKAQIWDTAGQERYRSITSAHYRRAVGALLVYDVTNRISFYN

CKKWLNELRASSYDDIVILLIANKIDLINKYNSNEKENVKENVGSQENKD

TVQEINDVDVVLMIEGMEFANENNLYFFEASAVTGYNVKEIFEFLIQQIY

NLKSRLPSIRDSNTLNTLTNNDFTNKNFNTNLSSKNFTGTENFTGTKNLE

KNSRQFGCLEQNCLTNNCIIQ

>TpRab11B

MADEDYEHVYKIILLGDATVGKSHLLCRYIRGDLPVQAKATIGVEFATRT

VPLASGGSVKAQIWDTAGQERYRSITSAHYRRAVGALLVYDVTNRSSFYN

CKKWLDELRASSYDDIVILLIANKVDLTNTQEGDNVRLDVVMAVEGMEFA

SENNLHFFEASAVTGYNVKEIFEFLIQQIYNLKSRLPSVTERITSFNSIV

PNLHTDKINKRDKLTQLGCLEQGCFNTNCNTQ
