# Supplementary material for: Comparative genomics of the Rab protein family in Apicomplexan parasites
Source: Microbes Infect. 2008 Apr;10(5):462–70. doi: 10.1016/j.micinf.2008.01.017 (PMC3317772; doi:10.1016/j.micinf.2008.01.017)
Supplement: Supplementary file 4 [file mmc4.doc]

TpRab1A -------------MCSFPGSFS-----------PLSLVIISSNSFSSVIVALESPVCCSD

TaRab1A -------------MCFFPTPFS-----------PFSLVIISSNSFSSAIVALENLAYCSD

PbRab1A -------------MNENRSRD---------YDYLYKIILIGDSGVGKSCILLRFS----D

PfRab1A -------------MTENRSRD---------YDYLYKIILIGDSGVGKSCILLRFS----D

TgRab1A MRLWWLARASLRPTRGNWPLDRVQLCARIPLDHLFKLVLIGDSGVGKSCLLLRFS----D

ChRab1A -------------MSAVRQKE----------DFLFKLVLIGDSGVGKSCLLLRFA----D

CpRab1A -------------MSAVRQKEY---------DFLFKLVLIGDSGVGKSCLLLRFAQ---D

BbRab1A -------------MVSRAAKD---------YDHLFKLVLIGDSGVGKSCVLLRFA----D

ChRab6 -------------MADNSSRNQ---------ISKFKFVFLGEQSVGKTSIITRFMY----

CpRab6 -------------MADNSSRNQ---------ISKFKFVFLGEQSVGKTSIITRFMY----
